# Disentangling the Effects of Ocean Carbonation and Acidification on Elemental Contents and Macromolecules of the Coccolithophore *Emiliania huxleyi*

**DOI:** 10.3389/fmicb.2021.737454

**Published:** 2021-10-20

**Authors:** Emei Xie, Kui Xu, Zhengke Li, Wei Li, Xiangqi Yi, Hongzhou Li, Yonghe Han, Hong Zhang, Yong Zhang

**Affiliations:** ^1^College of Environmental Science and Engineering, Fujian Key Laboratory of Pollution Control and Resource Recycling, Fujian Normal University, Fuzhou, China; ^2^Hubei Key Laboratory of Edible Wild Plants Conservation and Utilization, Hubei Engineering Research Center of Special Wild Vegetables Breeding and Comprehensive Utilization Technology, College of Life Sciences, Hubei Normal University, Huangshi, China; ^3^School of Food and Biological Engineering, Shanxi University of Science and Technology, Xi’an, China; ^4^College of Life and Environmental Sciences, Huangshan University, Huangshan, China; ^5^State Key Laboratory of Marine Environmental Science, College of Ocean and Earth Sciences, Xiamen University, Xiamen, China

**Keywords:** biomacromolecules, calcification, carbonate chemistry, coccolithophore, elemental contents, photosynthesis

## Abstract

Elemental contents change with shifts in macromolecular composition of marine phytoplankton. Recent studies focus on the responses of elemental contents of coccolithophores, a major calcifying phytoplankton group, to changing carbonate chemistry, caused by the dissolution of anthropogenically derived CO_2_ into the surface ocean. However, the effects of changing carbonate chemistry on biomacromolecules, such as protein and carbohydrate of coccolithophores, are less documented. Here, we disentangled the effects of elevated dissolved inorganic carbon (DIC) concentration (900 to 4,930μmolkg^−1^) and reduced pH value (8.04 to 7.70) on physiological rates, elemental contents, and macromolecules of the coccolithophore *Emiliania huxleyi*. Compared to present DIC concentration and pH value, combinations of high DIC concentration and low pH value (ocean acidification) significantly increased pigments content, particulate organic carbon (POC), and carbohydrate content and had less impact on growth rate, maximal relative electron transport rate (*rETR*_max_), particulate organic nitrogen (PON), and protein content. In high pH treatments, elevated DIC concentration significantly increased growth rate, pigments content, *rETR*_max_, POC, particulate inorganic carbon (PIC), protein, and carbohydrate contents. In low pH treatments, the extents of the increase in growth rate, pigments and carbohydrate content were reduced. Compared to high pH value, under low DIC concentration, low pH value significantly increased POC and PON contents and showed less impact on protein and carbohydrate contents; however, under high DIC concentration, low pH value significantly reduced POC, PON, protein, and carbohydrate contents. These results showed that reduced pH counteracted the positive effects of elevated DIC concentration on growth rate, *rETR*_max_, POC, PON, carbohydrate, and protein contents. Elevated DIC concentration and reduced pH acted synergistically to increase the contribution of carbohydrate–carbon to POC, and antagonistically to affect the contribution of protein–nitrogen to PON, which further shifted the carbon/nitrogen ratio of *E. huxleyi*.

## Introduction

Coccolithophores are an important group of the marine phytoplankton and characterized by production of particulate inorganic carbon (PIC) *via* calcification. Coccolithophores play a central role in the marine carbon cycle and contribute 1–10% to marine organic carbon fixation through photosynthesis (Poulton et al., 2007) and 40–60% of CaCO_3_ export to tropical and high-latitude sediments through calcification (Broecker and Clark, 2009). The cosmopolitan coccolithophore *Emiliania huxleyi* is thought to be the most representative phytoplankton species in modern oceans and can form massive blooms in temperate and sub-polar waters with cell concentrations up to 10×10^7^ cells L^−1^ (Tyrell and Merico, 2004; Kondrik et al., 2019; Kubryakova et al., 2021). The importance of coccolithophores is well investigated in the field of biogeochemistry due to their effects on the biogeochemical cycling of carbon (Monteiro et al., 2016; Feng et al., 2018). However, only a few studies report the biochemical basis for varying elemental composition of coccolithophores under changing seawater carbonate chemistry (Jones et al., 2013; Heidenreich et al., 2019; Zhang et al., 2021). Therefore, it is of interest to investigate the shifts in biochemical composition, such as protein, carbohydrate, lipid, and pigment of coccolithophores under various seawater carbonate chemistry conditions, which will improve our biochemical understanding on the contribution of this important phytoplanktonal function group to the marine biogeochemical cycle of carbon and nitrogen.

In recent years, calcification and photosynthesis of *E. huxleyi* received considerable attention with a number of studies investigating their responses to ocean acidification (OA; Meyer and Riebesell, 2015; Feng et al., 2017). Ocean acidification refers to a reduction in the pH of the surface ocean and an increase in dissolved inorganic carbon (DIC) and CO_2_ concentrations, caused primarily by uptake of anthropogenically derived CO_2_ from the atmosphere (Caldeira and Wickett, 2003). The studies showed that calcification rates of *E. huxleyi* were generally reduced and photosynthetic carbon fixation rates were increased under ocean acidification at the end of this century (Riebesell and Tortell, 2011; Feng et al., 2017). The microarray-based transcriptome profiling reveals that the observed lowered calcification under OA can be caused by impaired signal-transduction and ion–transport associated with Ca^2+^ and H^+^ fluctuation, and that the observed increases in organic carbon fixation may be attributed to stimulated carbon allocation to lipids under OA (Rokitta et al., 2012). In addition, the metabolome profiling reports minor changes in lipids, amino acids, and pigments of *E. huxleyi* in response to OA (Heidenreich et al., 2019). The proteome profiling also shows no-significant changes in most of the examined protein groups associated with many key metabolic processes of *E. huxleyi* under OA condition (Jones et al., 2013). One of the reasons for the biased conclusions introduced by transcriptome and proteome techniques may be due to non-homogeneous translation of all ribonucleic acids and the post-translational regulation of enzymatic machinery (Fernie and Stitt, 2012). To the best of our knowledge, few studies investigated the regulation of OA on both cellular elemental contents and biomacromolecules of coccolithophores.

Carbon dioxide concentration and pH are thought to affect coccolithophores in different ways. CO_2_ concentration mainly alters photosynthetic carbon fixation (Bach et al., 2013; Zhang et al., 2015), whereas pH affects cellular pH homeostasis, ion balance and hence, the synthesis of the bioactive products, such as enzymes and ion transporters (Monteiro et al., 2009; Taylor et al., 2011, 2017). Some studies have reported responses of PIC, particulate organic carbon (POC) and particulate organic nitrogen (PON) of *E. huxleyi* to changing CO_2_ and pH (Bach et al., 2013; Kottmeier et al., 2016; Zhang et al., 2021). However, little has been documented on the shifts in biomacromolecules of *E. huxleyi* under changing seawater carbonate chemistry, and it is still unclear how the shifts in biomacromolecules affect elemental composition of *E. huxleyi*. In the present work, we investigated variations in growth rate, electron transport rate, cellular PIC, POC, PON, protein, and carbohydrate contents of *E. huxleyi* under 400μatm CO_2_ and pH 8.04 treatment, under 400μatm CO_2_ and pH 7.70 treatment, under 1,000μatm CO_2_ and pH 8.04 treatment, and under 1,000μatm CO_2_ and pH 7.70 treatment and reported the contributions of protein–carbon and carbohydrate–carbon to POC and protein–nitrogen to PON in *E. huxleyi* under different carbonate chemistry conditions.

## Materials and Methods

### Experimental Setup

*Emiliania huxleyi* strain RCC1266, originally isolated from shelf water around Ireland (49^o^30^ʹ^ N, 10^o^30^ʹ^ W), was obtained from the Roscoff algal culture collection. Cells were maintained in semi-continuous cultures in artificial seawater (ASW) media prepared according to Berges et al. (2001) without the addition of NaHCO_3_, with a salinity of 33psu, under 200μmol photons m^−2^ s^−1^ of photosynthetically active radiation (PAR; measured using a LI-190SA quantum sensor, Beijing Ligaotai Technology Co. Ltd., China) and a 12:12h light/dark cycle (light period: 8:00 to 20:00) and 20°C in a thermo-controlling incubator (GXZ, Dongnan Instrument Company). The DIC-free ASW media was enriched with 64μmolL^−1^ NO_3_^−^, 4μmolL^−1^ PO_4_^3−^, f/8 concentrations for trace metals and vitamins (Guillard and Ryther, 1962). To disentangle the effects of carbonate system parameters on elemental contents and biomacromolecules of *E. huxleyi*, calculated amounts of Na_2_CO_3_ (1.20molkg^−1^, filtered by PTFE filter, 0.22μm pore size, Filter-Bio, Nantong) and hydrochloric acid (HCl, 2.00molkg^−1^) were added stepwise into the media to achieve four different carbonate chemistry conditions (Table 1). Initial CO_2_ concentration and pH_Total_ (Total scale) value were set to 400μatm and 8.04 under low CO_2_ and high pH treatment (1980μmolkg^−1^ DIC, LCHpH, present DIC and pH treatment), 400μatm and 7.70 under low CO_2_ and low pH treatment (910μmolkg^−1^ DIC, LCLpH, reduced pH), 1,000μatm and 8.04 under high CO_2_ and high pH treatment (4,930μmolkg^−1^ DIC, HCHpH, ocean carbonation), and 1,000μatm and 7.70 under high CO_2_ and low pH treatment (2,160μmolkg^−1^ DIC, HCLpH, ocean acidification), respectively. In each condition, the ASW was put into the incubator at 20°C for 24h and then filtered (0.22μm pore size, Polycap 75 AS, Whatman) and carefully pumped into autoclaved 100ml, 1,120ml, and 2,350ml polycarbonate bottles with no headspace to minimize gas exchange. 100ml seawater was used to determine initial total alkalinity (TA) and pH_Total_ of seawater, 1,120ml bottles were used to acclimate cells to experimental conditions (one replicate), and the main experiment culture was conducted in 2350ml bottles (four replicates). The cells were inoculated to achieve an initial concentration of about 3,000 cell ml^−1^ and cultured in each experimental condition for 2days and then diluted to the initial cell density again (Supplementary Figure S1). This process was performed four times under each treatments, and cells were maintained in exponential growth phase for a minimum of 13 generations (Supplementary Table S1) and then transferred from 1,120ml to 2,350ml bottles at the same time. The volume of culture inoculum was calculated to match the volume of media taken out from the bottles before inoculation. Culture bottles were rotated 10 times until cells were mixed at 9:00, 14:00 and 18:00 (Beijing Time). In the second days of the main experiments, cell densities were lower than 70,000 cells ml^−1^ under all treatments, and sub-samples in each incubation bottle were harvested with different volume for measurements of TA, pH_Total_, cell density, total particulate carbon (TPC), POC, and nitrogen (PON), protein, carbohydrate, and pigment. It should be mentioned that Barcelos e Ramos et al. (2010) reported that *E. huxleyi* can rapidly alter the rates of essential metabolical processes in response to changing seawater carbonate chemistry within 26h. In this study, *E. huxleyi* cells were incubated under each treatment with 4 biological replicates for 2days, thus the results found here were comparable with other studies (see discussion section; Feng et al., 2017; Bi et al., 2018).

**Table 1:** Carbonate chemistry parameters at the start and end of the incubation, and changes in carbonate chemistry parameters during the incubation.

		*p*CO_2_	pH	TA	DIC	$HCO_{3}^{-}$	$CO_{3}^{2-}$	CO_2_
		(μatm)	(total scale)	(μmolkg^−1^)	(μmolkg^−1^)	(μmolkg^−1^)	(μmolkg^−1^)	(μmolkg^−1^)
LCHpH	Start	405	8.04	2,236	1983	1786	183	13.2
(Present DIC and pH)	End	322±6	8.11±0.01	2,133±10	1854±10	1,647±11	196±2	10.5±0.2
	Change	20.61%	0.07	4.61%	6.51%	7.80%	7.09%	20.61%
LCLpH	Start	423	7.70	976	906	852	40	13.8
(Reduced pH)	End	339±7	7.73±0.01	847±6	776±7	728±6	36±1	11.1±0.2
	Change	19.82%	0.03	13.23%	14.42%	14.58%	9.00%	19.82%
HCHpH	Start	1,006	8.04	5,432	4,927	4,439	455	32.9
(Ocean carbonation)	End	880±49	8.08±0.02	5,229±17	4,702±33	4,205±46	468±16	28.7±1.6
	Change	12.50%	0.04	3.74%	4.57%	5.26%	2.73%	12.50%
HCLpH	Start	1,006	7.70	2,264	2,157	2029	95	32.9
(Ocean acidification)	End	901±14	7.72±0.01	2,162±17	2050±17	1925±15	96±2	29.4±0.4
	Change	10.44%	0.03	4.47%	4.97%	5.14%	1.17%	10.44%

Data are one replicate at the start of the incubation and means±*sd* of 4 replicates at the end of the incubation.

### Carbonate Chemistry Measurements

In the second days of the main experiments, to reduce impact of respiration of *E. huxleyi* on seawater pH value and total alkalinity (TA), 40ml and 50ml samples were, respectively, filtered (PTFE filter, 0.22μm pore size, Nantong) 5h after the onset of the light period (at 13:00) and used to measure the pH_Total_ and TA. The bottles were filled from bottom to top with overflow, and closed immediately without a headspace. The pH_Total_ was measured immediately at 20°C using a pH meter which was calibrated with buffers (Tris•HCl, Hanna) at pH 4.01, 7.00 and 10.00. The pH value was not corrected with a standard buffer of defined pH in seawater (Dickson et al., 2007). TA samples were stored at 4°C for a maximum of 7days (Zhang et al., 2021), and TA was measured at 20°C by potentiometric titration (AS-ALK1+, Apollo SciTech) according to Dickson et al. (2003). Phosphate concentration was not measured at the beginning and end of the incubations. In this study, the carbonate system was estimated from TA, pH_Total_, temperature, salinity, and phosphate (4μmolL^−1^) using the CO2SYS program (Pierrot et al., 2006) with carbonic acid constants, *K_1_* and *K_2_*, taken from Roy et al. (1993).

### Cell Density Measurements

Twenty fiveml samples were taken daily 6h after the onset of the light period (at 14:00) and fresh ASW with the same carbonate chemistry as in the initial treatment conditions were added as top-up. Cell density was measured using a Z2 Coulter Particle Count and Size Analyzer (Beckman Coulter). The diameter of detected particles was set to 3–7μm in the instrument, which excluded detached coccoliths (Müller et al., 2012). Cell concentration was also measured by microscopy (ZEISS), and variation in measured cell concentrations was±8% between the two methods. Growth rate (*μ*) was calculated for each replicate according to the equation: *μ*=(ln *N*_t_−ln *N*_0_)/*d*, where *N*_t_ and *N*_0_ refer to the cell concentrations in the second day and beginning of the main experiment, respectively, and *d* was the growth period in days.

### Chlorophyll Fluorescence Measurements

The photosynthetic fluorescence parameters were determined using a pulse amplitude modulated fluorometer (WATER–PAM, Walz, Effeltrich, Germany). 2ml samples were taken from the incubation bottles and kept in the dark for 15min at 20°C. The assay light levels (A-PAR) between 0 and 1,120μmol photons m^−2^ s^−1^ were applied in nine steps and 45s each in fast light response curve measurements. The instant minimal (*F*_0_ʹ) and maximal fluorescence (*F*_m_ʹ) were, respectively, determined at the end of each A-PAR and saturating light pulse (800ms, 3,000μmol photons m^−2^ s^−1^). The effective photochemical quantum yield (*Yield*) was calculated as: *Yield*=(*F*_m_ʹ−*F*_0_ʹ)/*F*_m_ʹ (Baker, 2008). The relative electron transport rate (*rETR*) was calculated as: *rETR*=*Yield*×A-PAR (Ralph and Gademann, 2005). The parameters of the photosynthesis vs. irradiance curves (P–I curves) were analyzed as follows: *rETR*=*rETR*_max_×tanh (*a*×A-PAR/*rETR*_max_; Jasby and Platt, 1976). The maximal relative electron transport rate (*rETR*_max_) represents the light-saturating level of *rETR*, and light use efficiency (*a*) was derived from the slope of each electron transport rate (*ETR*) vs. light curve. Saturating light intensity, *I*_k_, is calculated from the expression *rETR*_max_/*a* and is characteristic for the onset of light saturation.

### Pigment Measurements

One hundredml samples were filtered onto pre-combusted GF/F filters (at 450°C for 6h) with low vacuum pressure (<0.02MPa) at 15:00 and soaked in 5ml 90% acetone overnight at 4°C in the dark. The extracts were centrifuged at 4300×*g* for 10min to remove glass fibers. Absorbance (*A*) of the supernatant on 750, 664, 647, and 480nm was measured using a spectrophotometer (SP-722, Shanghai Spectrum Instruments, China). The chlorophyll *a* (Chl *a*) concentration was calculated as: Chl *a* (μgml^−1^)=−1.93×(*A*_647_–*A*_750_)+11.93×(*A*_664_–*A*_750_). The carotenoid concentration was calculated as: Carotenoid (μgml^−1^)=4×(*A*_480_–*A*_750_; Jeffrey and Humphrey, 1975; Davies, 1976).

### Total Particulate Carbon, Particulate Organic Carbon, and Nitrogen Measurements

Two hundredml samples for determinations of TPC and POC (PON) were, respectively, gently filtered onto GF/F filters (pre-combusted at 450°C for 6h) at the same time (15:30) in each treatment, rinsed three times with DIC–free ASW media, and then stored in the dark at −20°C. For determination of POC (PON), samples were fumed with HCl for 12h to remove inorganic carbon. TPC and POC (PON) samples were dried at 60°C for 12h and analyzed using an Elementar CHNS analyzer (Vario EL cube, GmbH, Germany). PIC quota was calculated as the difference between TPC and POC quota (Fabry and Balch, 2010). PIC, POC, and PON production rates were calculated by multiplying cellular contents with *μ* (d^−1^), respectively, (Supplementary Figure S2).

### Protein and Carbohydrate Measurements

After mixing, 700ml and 800ml samples for determinations of protein and carbohydrate were, respectively, filtered onto polycarbonate filters (25mm diameter, 0.6μm pore size, Nuclepore, Whatman) and onto pre-combusted GF/F filters at 15:30 and then stored in the dark at −80°C. Protein samples were put into 2ml MP Biomedical tubes (Lysing, Matrix D) containing large ceramic beads. After being freeze-dried, protein samples were extracted by a mixture of 0.7ml 1X protein extraction buffer (0.10mmolL^−1^ 4-(2-aminoethyl) benzenesulfonyl fluoride hydrochloride (protease inhibitor), 125.00mmolL^−1^ ethylene diamine tetraacetic acid, 28.07mmolL^−1^ Tris base, 21.10mmolL^−1^ Tris-HCl, 1085.89mmolL^−1^glycerol and 73.44mmolL^−1^ lithium dodecyl sulfate). Cells were lysed using a FastPrep–24 machine (MP Biomedicals, United States) at 6.5ms^−1^ with 4cycles and 1min each, and samples were chilled in an ice bath for 2min between 2cycles. Then, the samples were centrifuged at 10,000×*g* for 5min (Centrifuge 5,418 R, Eppendorf, Germany), and extracted protein in the supernatant was determined at 562nm using the BCA Assay with Bovine gamma globulin as a standard, using a spectrophotometer (Ni et al., 2016).

Carbohydrate samples were firstly treated with 12.00molL^−1^ sulfuric acid (H_2_SO_4_) in the dark for 1h and then diluted by milliQ water for a final H_2_SO_4_ concentration of 1.20molL^−1^. Samples were then sonicated for 5min and vortexed for 30s, and boiled at 90.0°C for 3h in a water bath (Pakulski and Benner, 1992). The concentration of monosaccharide was analyzed at 490nm by phenol–sulfuric reaction using a spectrophotometer with D-glucose as standard (Masuko et al., 2005).

### Data Analysis

The elemental content of macromolecular pools was calculated based on a mean elemental stoichiometry for protein, carbohydrates, Chl *a* and carotenoid (Geider and LaRoche, 2002). Two-way ANOVA were used to determine the main effects of CO_2_ concentration and pH value and their interactions on each variable. Tukey *post hoc* tests were conducted to identify significant differences between two CO_2_ concentrations or two pH values. Normality of residuals was tested using a Shapiro–Wilk test, and a Levene test was used to analyze for homogeneity of variances. All statistical calculations were performed using R Core Team (2018) with the packages carData, lattice, and nlme.

## Results

### Carbonate Chemistry

Initial CO_2_ concentrations ranged from 400 to 420μatm under low DIC (LC) treatments and were 1,010μatm under high DIC (HC) treatments (Table 1). Initial pH values were 8.04 and 7.70 in high and low pH conditions, respectively. During the experiment, DIC concentrations decreased by, on average, 4.57 to 14.42% under various carbonate chemistry treatments. Correspondingly, CO_2_ concentrations decreased by, on average, 10.44 to 20.61%, and pH_Total_ values increased by less than 0.07 under different experimental treatments (Table 1). Under low pH treatment, low DIC concentration (LCLpH) represents low CO_2_, bicarbonate (HCO_3_^−^), carbonate (CO_3_^2−^) and TA concentrations, and under high pH treatment, high DIC concentration (HCHpH) represents high CO_2_, HCO_3_^−^, CO_3_^2−^, and TA concentrations (Table 1).

### Growth Rate, Cellular Pigment Content, and Photosynthetic Fluorescence Parameter

**Figure 1:**
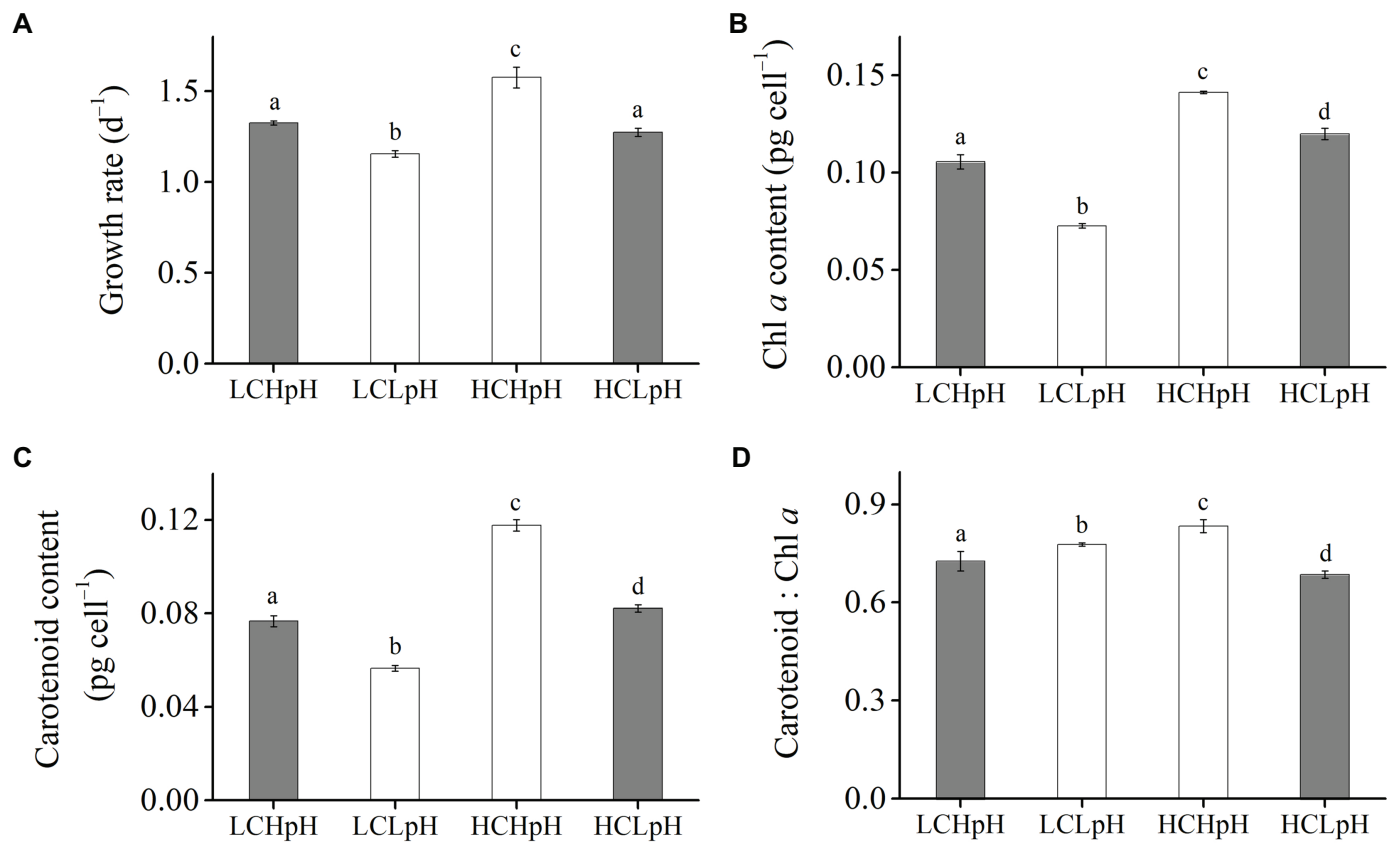
**(A)** Growth rate, **(B)** chlorophyll *a* (Chl *a*) content, **(C)** carotenoid content and **(D)** carotenoid: Chl *a* ratio of *Emiliania huxleyi* RCC1266 under present DIC concentration and high pH value (LCHpH, gray), low DIC concentration and low pH value (LCLpH, reduced pH), high DIC concentration and high pH value (HCHpH, ocean carbonation), and high DIC concentration and low pH value (HCLpH, ocean acidification, gray) treatments. Data were obtained after cells were acclimated to experimental conditions for at least 13 generations and means±standard deviation (SD) of 4 replicate populations. Different letters (a, b, c, d) in each panel represent significant differences between four treatments (Tukey *Post hoc*, *p*<0.05).

**Figure 2:**
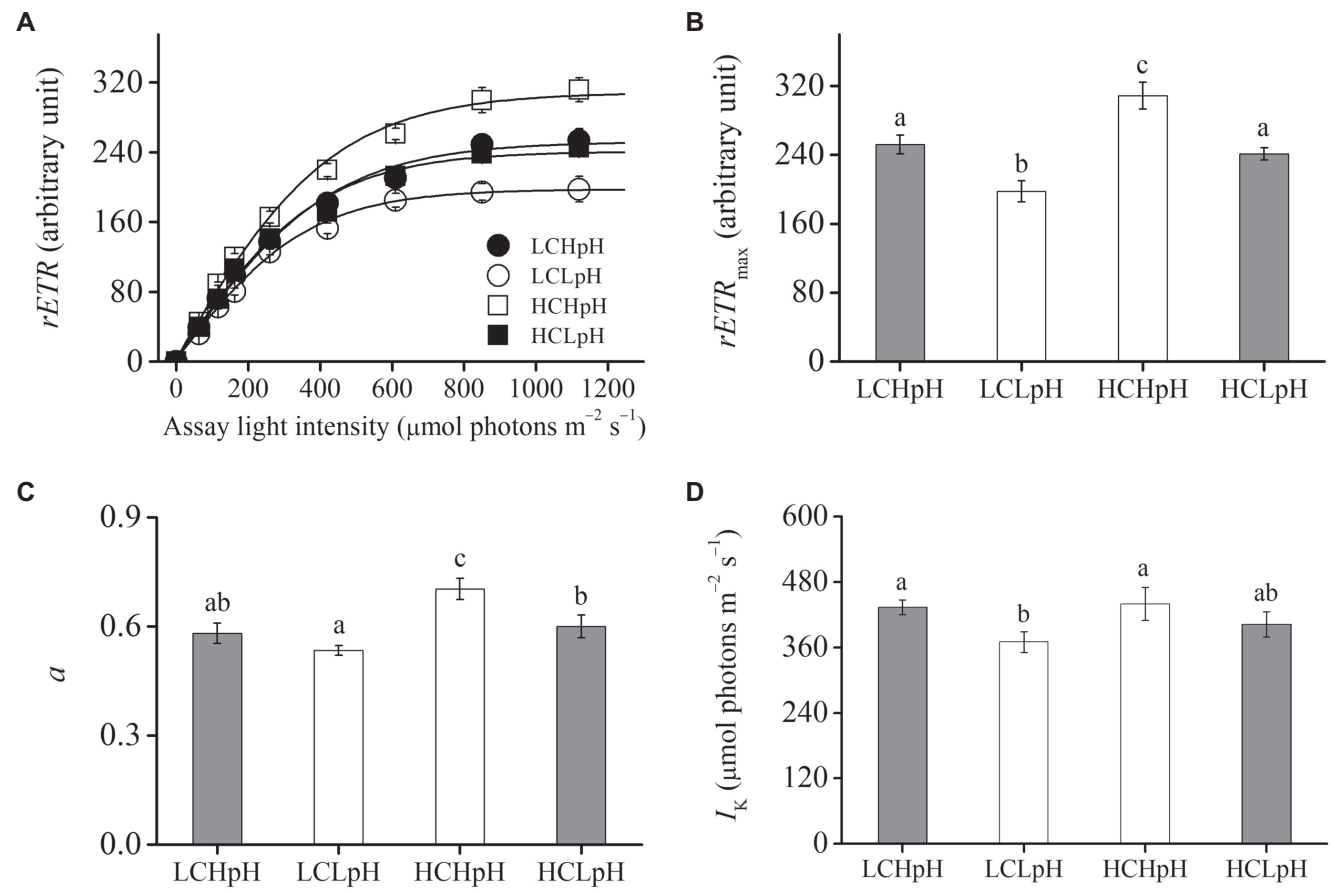
**(A)** Relative electron transport rate (*rETR*) as a function of the assay light intensity (0–1,120μmol photons m^−2^ s^−1^), **(B)** maximal relative electron transport rate (*rETR*_max_), **(C)** light use efficiency (*a*) and **(D)** saturating light intensity (*I*_k_) of *E. huxleyi* RCC1266 under the LCHpH (gray), LCLpH, HCHpH, and HCLpH (gray) treatments. Different letters (a, b, c) in each panel represent significant differences between four treatments (Tukey *Post hoc*, *p*<0.05). For more information, please see Figure 1.

Compared to present DIC concentration and pH value (LCHpH), ocean acidification (high DIC concentration and low pH value, HCLpH) did not significantly affect growth rate, *rETR*_max_, light use efficiency (*a*), and saturating light intensity (*I*_k_; all *p*>0.10) and increased chlorophyll *a* (Chl *a*) and carotenoid content by 13.56 and 7.17%, respectively (both *p*<0.01; Figures 1, 2, gray). Under the same pH treatments, the effects of elevated DIC concentration on growth rate, Chl *a* and carotenoid contents, *rETR*_max_, *a*, and *I*_k_ were positive, which can be seen by comparing these variables at HC concentrations with their paired LC concentrations (Figures 1, 2). Compared to low DIC concentration, high DIC concentration increased growth rate by 18.96% at high pH and by 10.34% at low pH, increased Chl *a* content by 33.83% at high pH and by 65.02% at low pH, increased carotenoid content by 53.55% at high pH and by 45.51% at low pH (Figures 1A–C), increased *rETR*_max_ by 22.50% at high pH and by 21.99% at low pH, increased *a* by 20.88% at high pH and by 12.40% at low pH (all *p*<0.05), respectively (Figures 2B,C; Tables 2, 3) and did not significantly increase *I*_k_ under both high and low pH treatments (both *p*>0.10; Figure 2D).

**Table 2:** Results of two-way ANOVAs of the effects of dissolved inorganic carbon (DIC) and pH and their interaction on growth rate, chlorophyll *a* (Chl *a*) and carotenoid contents, carotenoid: Chl ratio, *rETR*_max_, light use efficiency (*a*), saturating light intensity (*I*_k_), PIC, POC and PON contents, PIC/POC and POC/PON ratios, protein and carbohydrate contents, the percentages of protein–carbon to POC (Protein–C: POC), carbohydrate–carbon to POC (carbohydrate–C: POC), Chl–carbon to POC (Chl–C: POC) and carotenoid–carbon to POC (Carotenoid–C: POC), and the percentages of protein–nitrogen to PON (Protein–N: PON) and Chl–nitrogen to PON (Chl–N: PON).

	DIC		pH		DIC×pH	
	*F*	*p*	*F*	*p*	*F*	*p*
Growth rate	130.12	<0.01	210.44	<0.01	16.43	<0.01
Chl *a*	1199.04	<0.01	514.14	<0.01	23.02	<0.01
Carotenoid	1130.68	<0.01	787.43	<0.01	59.55	<0.01
Carotenoid: Chl ratio	0.61	=0.45	26.25	<0.01	108.56	<0.01
*rETR*_max_	71.93	<0.01	106.83	<0.01	1.26	=0.28
*a*	50.23	<0.01	32.12	<0.01	4.34	=0.06
*I*_k_	2.95	=0.11	20.32	<0.01	1.36	=0.27
PIC content	75.39	<0.01	413.59	<0.01	4.61	=0.05
POC content	79.64	<0.01	7.90	=0.02	117.30	<0.01
PON content	7.54	=0.02	0.01	=0.92	102.05	<0.01
PIC/POC ratio	2.96	=0.11	199.08	<0.01	14.09	<0.01
POC/PON ratio	9.92	<0.01	1.96	=0.19	0.56	=0.47
Protein content	200.44	<0.01	138.93	<0.01	339.12	<0.01
Carbohydrate content	248.18	<0.01	4.06	=0.07	26.01	<0.01
Protein–C: POC	2.06	=0.18	27.12	<0.01	6.12	=0.03
Carbohydrate–C: POC	34.24	<0.01	0.32	=0.58	12.58	<0.01
Chl–C: POC	51.64	<0.01	52.22	<0.01	104.76	<0.01
Carotenoid–C: POC	63.31	<0.01	106.40	<0.01	27.66	<0.01
Protein–N: PON	34.84	<0.01	50.75	<0.01	5.58	=0.04
Chl–N: PON	199.78	<0.01	121.14	<0.01	201.95	<0.01

**Table 3:** Summary of all parameters under four carbonate chemistry treatments.

	LCHpH	LCLpH	HCHpH	HCLpH
	(Present DIC and pH)	(Reduced pH)	(Ocean carbonation)	(Ocean acidification)
Growth rate (d^−1^)	1.33 ± 0.01^a^	*1.16 ± 0.02^b^*	1.58 ± 0.06^c^	1.27 ± 0.02^a^
Chl *a* (pg cell^−1^)	0.11 ± 0.01^a^	*0.07 ± 0.01^b^*	0.14 ± 0.01^c^	0.12 ± 0.01^d^
Carotenoid (pg cell^−1^)	0.08 ± 0.02^a^	*0.06 ± 0.01^b^*	0.12 ± 0.01^c^	0.08 ± 0.01^d^
Car: Chl ratio	0.73 ± 0.03^a^	0.78 ± 0.01^b^	0.83 ± 0.02^c^	*0.69 ± 0.01^d^*
*rETR*_max_ (arbitrary unit)	252.14 ± 10.84^a^	*197.71 ± 12.10^b^*	308.87 ± 15.57^c^	241.18 ± 7.21^a^
*a*	0.58 ± 0.03^ab^	0.53 ± 0.01^a^	0.70 ± 0.03^c^	0.60 ± 0.031^b^
*I*_k_ (μmol photons m^−2^ s^−1^)	433.78 ± 13.68^a^	*370.10 ± 19.12^b^*	439.98 ± 30.39^a^	402.49 ± 23.21^ab^
PIC content (pg C cell^−1^)	4.17 ± 0.29^a^	*1.80 ± 0.29^b^*	5.58 ± 0.27^c^	*2.65 ± 0.18^d^*
POC content (pg C cell^−1^)	10.06 ± 0.29^a^	11.87 ± 0.16^b^	14.53 ± 0.70^c^	11.44 ± 0.46^b^
PON content (pg N cell^−1^)	1.68 ± 0.06^a^	2.13 ± 0.15^b^	2.25 ± 0.07^b^	1.81 ± 0.05^a^
PIC/POC ratio	0.42 ± 0.04^a^	*0.15 ± 0.02^b^*	0.38 ± 0.03^a^	*0.23 ± 0.02^c^*
POC/PON ratio	6.00 ± 0.36^ab^	5.59 ± 0.41^a^	6.47 ± 0.48^b^	6.34 ± 0.26^ab^
Protein (pg cell^−1^)	5.76 ± 0.23^a^	6.28 ± 0.52^a^	9.24 ± 0.11^b^	5.86 ± 0.42^a^
Carbohydrate (pg cell^−1^)	3.34 ± 0.18^a^	3.55 ± 0.26^a^	5.19 ± 0.18^b^	4.61 ± 0.17^c^
Protein–C: POC (%)	30.39 ± 1.88^ab^	28.02 ± 2.09^a^	33.76 ± 1.27^b^	27.13 ± 1.54^a^
Carbohydrate–C: POC (%)	13.28 ± 0.87^ab^	11.96 ± 1.00^a^	14.31 ± 0.87^bc^	16.14 ± 0.81^c^
Chl–C: POC (%)	0.78 ± 0.05^a^	*0.45 ± 0.01^b^*	0.72 ± 0.04^a^	0.78 ± 0.04^a^
Carotenoid–C: POC (%)	0.61 ± 0.02^a^	*0.38 ± 0.01^b^*	0.65 ± 0.04^a^	0.58 ± 0.03^a^
Protein–N: PON (%)	54.93 ± 1.60^a^	*47.27 ± 4.93^b^*	65.76 ± 2.58^c^	51.92 ± 3.59^ab^
Chl–N: PON (%)	0.40 ± 0.01^a^	*0.22 ± 0.02^b^*	0.40 ± 0.01^a^	0.42 ± 0.02^a^

Different letters (a, b, c, d) represent significant differences between four treatments (Tukey *post hoc*, *p*<0.05). Values in italic indicate significant lower and those in bold indicate significant larger under the LCLpH (reduced pH), HCHpH (ocean carbonation), and HCLpH (ocean acidification) treatments in comparison with the LCHpH (present DIC and pH) treatment. For more information, please see Table 2.

Under the same CO_2_ concentrations, the effects of reduced pH value on growth rate, Chl *a* and carotenoid contents, *rETR*_max_, *a* and *I*_k_ were negative, which can be seen by comparing these variables in low pH condition with their paired high pH condition (Figures 1, 2). Compared to high pH value, low pH value reduced growth rate by 12.82% at LC and by 19.14% at HC, reduced Chl *a* content by 31.18% at LC and by 15.14% at HC, reduced carotenoid content by 26.35% at LC and by 30.21% at HC (Figures 1A–C), reduced *rETR*_max_ by 21.59% at LC and by 21.91% at HC (all *p*<0.01), reduced *a* by 8.16% at LC and by 14.60% at HC (*p*=0.10 at LC; *p*<0.01 at HC) and reduced *I*_k_ by 14.68% at LC and by 8.52% at HC (*p*<0.01 at LC; *p*=0.14 at HC), respectively (Figures 2B–D). These results showed that elevated DIC concentration compensated for the negative effects of reduced pH on growth rate, Chl *a* and carotenoid contents, *rETR*_max_, *a* and *I*_k_.

### Cellular PIC, POC, and PON Contents, PIC/POC Ratio and POC/PON Ratio

**Figure 3:**
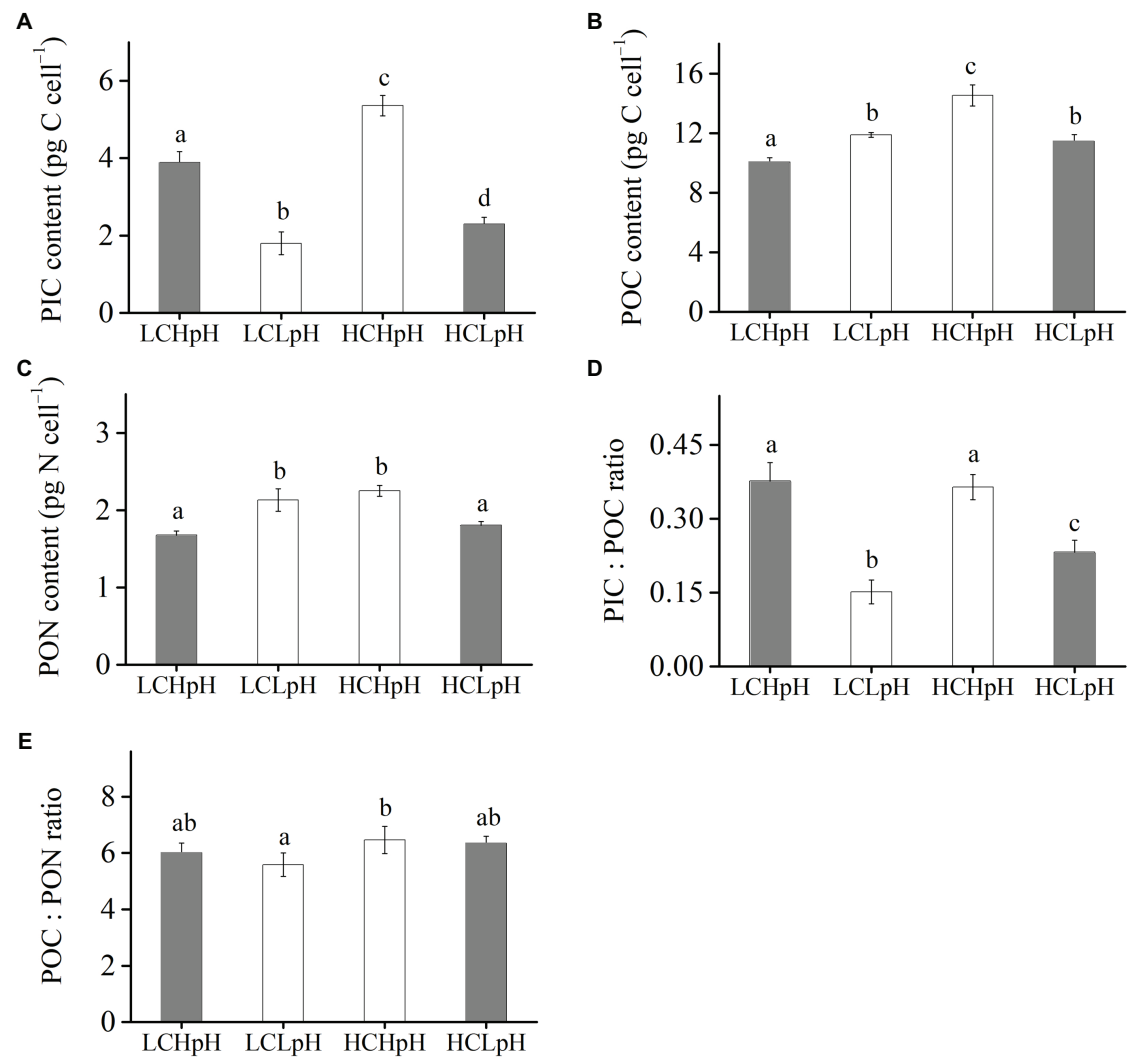
**(A)** Particulate inorganic carbon (PIC) content, **(B)** particulate organic carbon (POC) content, **(C)** particulate organic nitrogen (PON) content, **(D)** PIC/POC ratio, and **(E)** POC/PON ratio of *E. huxleyi* RCC1266 under the LCHpH (gray), LCLpH, HCHpH, and HCLpH (gray) treatments. Different letters (a, b, c, d) in each panel represent significant differences between different treatments (Tukey *Post hoc*, *p*<0.05). For more information, please see Figure 1.

Compared to present DIC concentration and pH value (LCHpH), ocean acidification (HCLpH) reduced PIC content by 40.93% and PIC/POC ratio by 44.05%, and increased POC content by 13.75% and did not significantly affect PON content (*p*=0.24) and POC/PON ratio (*p*=0.62; Figure 3, gray). The effects of elevated DIC concentration on cellular PIC, POC, and PON contents, and on PIC/POC ratio and POC/PON ratio were pH dependent (Figure 3). In high pH condition, elevated DIC concentration increased PIC content by 37.93%, POC content by 44.43%, PON content by 34.09% (all *p*<0.01; Tables 2, 3) and did not significantly affect PIC/POC ratio and POC/PON ratio. In low pH condition, elevated DIC concentration increased PIC content (*p*<0.01), PIC/POC ratio (*p*=0.01) and POC/PON ratio (*p*=0.07) did not significantly affect POC content and significantly reduced PON content.

Under both low and high DIC concentrations, reduced pH value reduced PIC contents by 53.74–57.17% and PIC/POC ratios by 39.62–63.53% (Figures 3A,D). The effects of reduced pH value on POC and PON contents were DIC dependent (Figures 3B,C). In low DIC concentration, reduced pH value increased POC content by 18.03% and PON content by 27.09% (both *p*<0.01); in high DIC concentration, it reduced POC content by 21.24% and PON content by 19.78% (both *p*<0.01; Figures 3B,C). These results showed that reduced pH value mainly reduced PIC content and PIC/POC ratio and reduced pH value and elevated DIC concentration acted antagonistically to affect cellular POC and PON contents.

### Cellular Protein and Carbohydrate Contents

**Figure 4:**
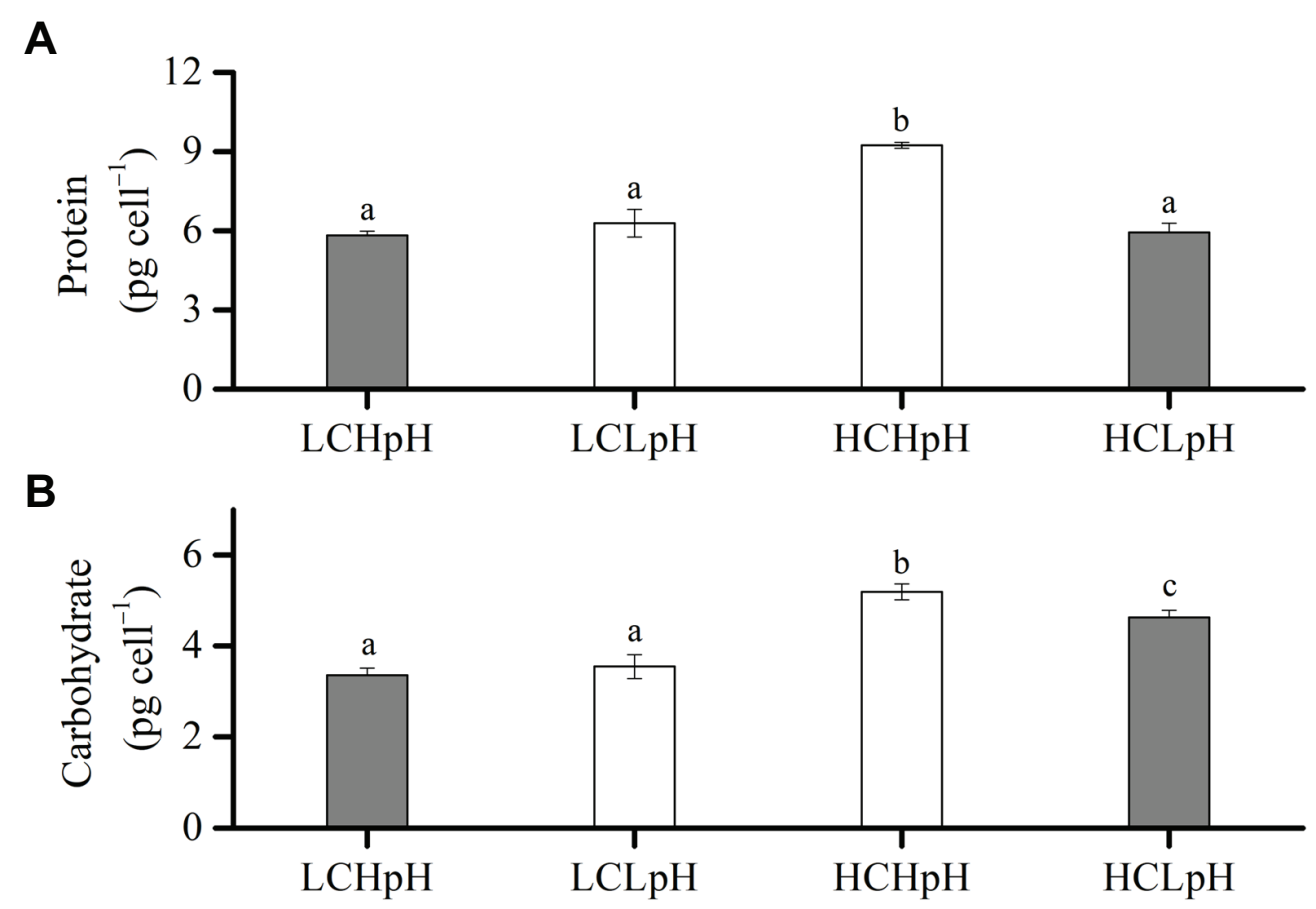
**(A)** Cellular protein content and **(B)** cellular carbohydrate content of *E. huxleyi* RCC1266 under the LCHpH (gray), LCLpH, HCHpH, and HCLpH (gray) treatments. Different letters (a, b, c) in each panel represent significant differences between four treatments (Tukey *Post hoc*, *p*<0.05). For more information, please see Figure 1.

Compared to present DIC concentration and pH value (LCHpH), ocean acidification (HCLpH) did not significantly affect protein content and increased carbohydrate content by 38.20% (Figure 4, gray). The effect of elevated DIC concentration on cellular protein content was pH dependent (Figure 4A). In high pH condition, elevated DIC concentration increased protein content by 60.42%, and in low pH condition, it did not significantly affect protein content (*p*=0.06). In low CO_2_ concentration, reduced pH value did not significantly affect protein content (*p*=0.05), and in high CO_2_ concentration, it significantly reduced protein content (*p*<0.01).

The effect of elevated DIC concentration on carbohydrate content was positive, which can be seen by comparing the carbohydrate contents in high DIC concentrations with their paired low DIC concentrations (Figure 4B). Elevated DIC concentration increased carbohydrate content by 55.43% in high pH condition and by 30.02% in low pH condition. The effect of reduced pH value on carbohydrate content was DIC concentration dependent. Reduced pH value did not significantly affect carbohydrate content in low DIC concentration and reduced carbohydrate content by 11.08% in high DIC concentration (Figure 4B, *p*<0.01). These results showed that elevated DIC concentration increased protein content in high pH condition but not in low pH condition and mainly increased carbohydrate content under both low and high pH treatments (Figures 4A,B).

### Percentage of POC Allocated to Protein and Carbohydrate, and Percentage of PON Allocated to Protein, and Their Correlation With Growth Rate

**Figure 5:**
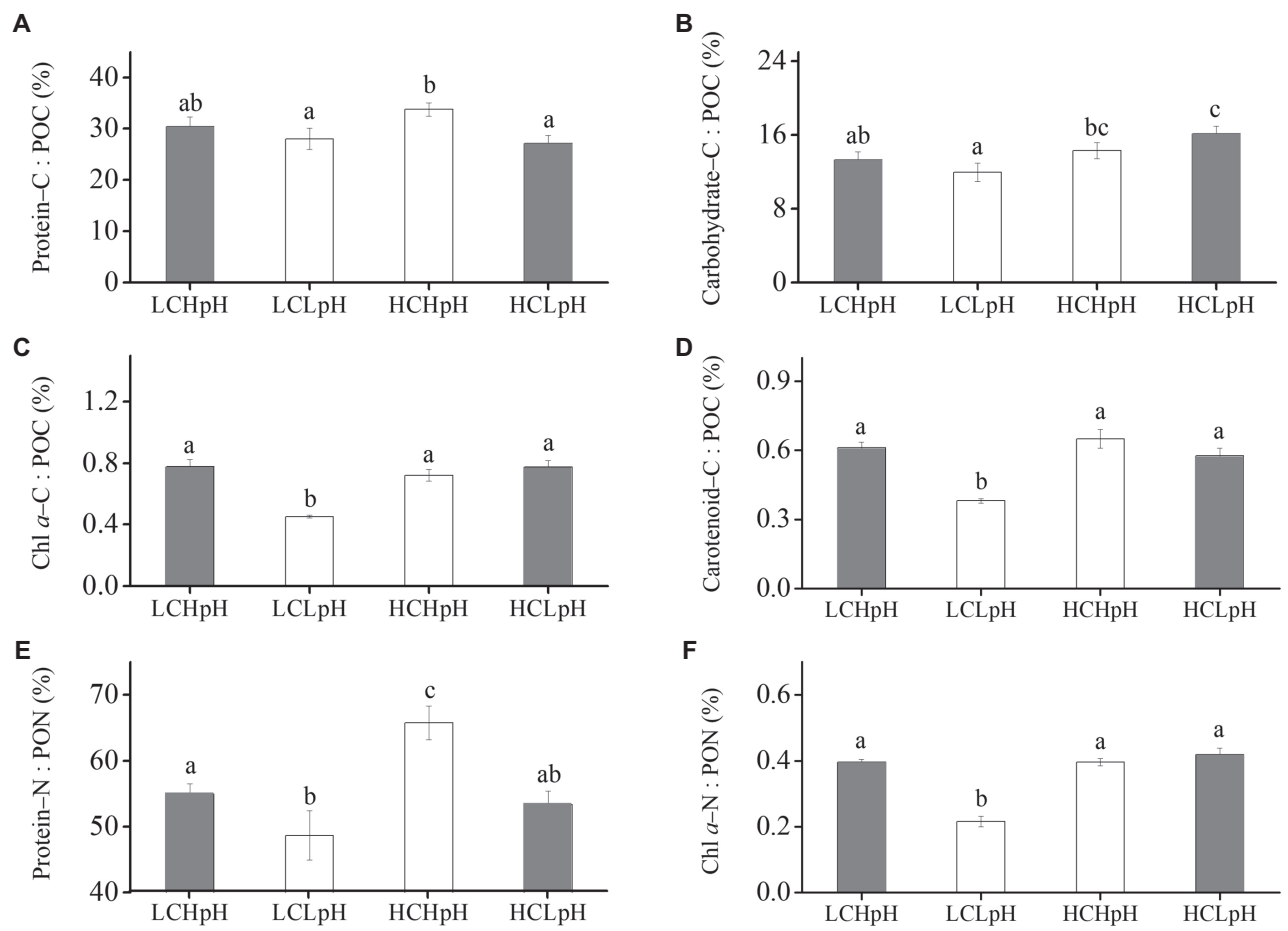
Percentage of POC allocated to **(A)** protein, **(B)** carbohydrate, **(C)** Chl *a* and **(D)** carotenoid, percentage of PON allocated to **(E)** protein and **(F)** Chl *a* of *E. huxleyi* RCC1266 under the LCHpH (gray), LCLpH, HCHpH, and HCLpH (gray) treatments. Different letters (a, b, c) in each panel represent significant differences between four treatments (Tukey *Post hoc*, *p*<0.05). For more information, please see Figure 1.

Compared to present DIC concentration and pH value (LCHpH), ocean acidification (HCLpH) did not significantly reduce the percentage of POC allocated to protein (*p*=0.08; Figure 5A, gray), significantly increased the percentage of POC allocated to carbohydrate by 21.52% (Figure 5B, gray) and did not significantly affect the percentage of PON allocated to protein (*p*=0.82; Figure 5E, gray). Under high or low pH treatments, elevated DIC concentrations did not significantly affect the percentages of POC allocated to protein (Protein–C: POC; *p*=0.07 at high pH; *p*=0.88 at low pH; Figure 5A; Tables 2, 3). Reduced pH did not significantly affect the percentages of POC allocated to protein in low DIC concentration (*p*=0.27) and significantly reduced this percentage in high DIC concentration.

Elevated DIC concentration did not show significant influence on the percentage of POC allocated to carbohydrate (Carbohydrate–C: POC) under high pH treatment (*p*=0.40) and significantly increased this percentage by 34.98% under low pH treatment (Figure 5B). Under low or high DIC concentrations, reduced pH value did not significantly affect the percentages of POC allocated to carbohydrate (*p*=0.21 at LC; *p*=0.06 at HC). The percentages of POC allocated to Chl *a* (Chl *a*–C: POC) and carotenoid (Carotenoid–C: POC) were 0.45–0.78% and 0.38–0.65%, respectively, under different carbonate chemistry treatments (Figures 5C,D).

Under high or low pH treatments, the effect of elevated DIC concentration on the percentage of PON allocated to protein (Protein–N: PON) was positive, which can be seen by comparing this percentage in high DIC concentrations with their paired low DIC concentrations (Figure 5E). Elevated DIC concentration increased the percentage of PON allocated to protein by 19.73% in high pH condition and by 9.86% in low pH condition (*p*=0.11). Under low or high DIC concentrations, the effect of reduced pH value on percentage of PON allocated to protein was negative, which can be seen by comparing this percentage in low pH conditions with their paired high pH conditions (Figure 5E). Reduced pH value reduced the percentage of PON allocated to protein by 13.95% in low DIC concentration and by 21.04% in high DIC concentration (both *p*<0.05). These results showed that elevated DIC concentration and reduced pH value acted synergistically to increase the percentage of POC allocated to carbohydrate (Figure 5B), and antagonistically to affect the percentage of PON allocated to protein (Figure 5E).

**Figure 6:**
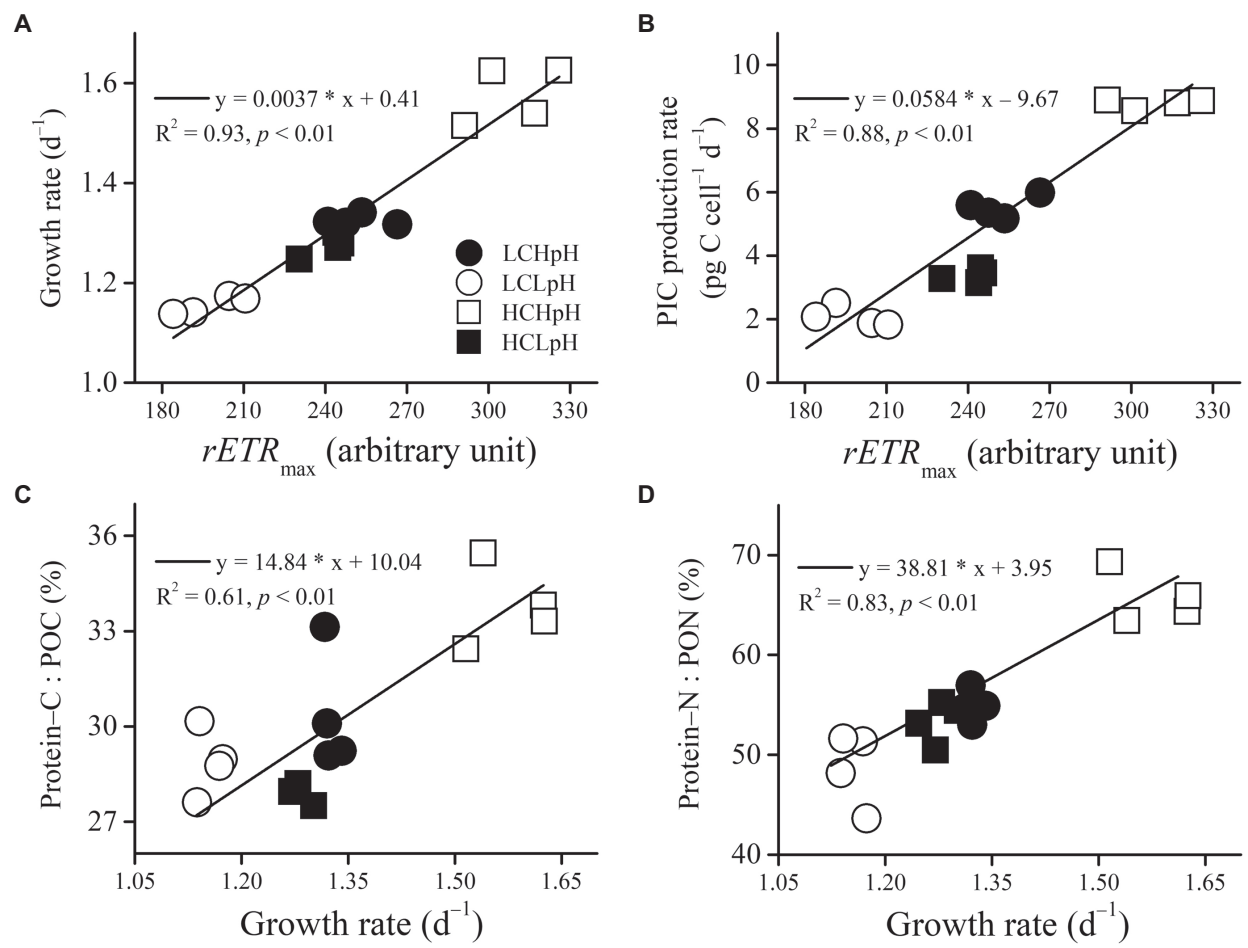
Relation between *rETR*_max_ and **(A)** growth rate or **(B)** PIC production rate, and relation between growth rate and **(C)** percentage of POC allocated to protein or **(D)** percentage of PON allocated to protein of *E. huxleyi* RCC1266. Line in each panel was fitted based on data of four independent cultures under each treatment condition. For more information, please see Figure 1.

Under four carbonate chemistry treatments, growth rate or PIC production rate significantly and positively correlated with *rETR*_max_ (R^2^=0.93 and 0.88, both *p*<0.01; Figures 6A,B). In addition, the percentage of POC allocated to protein (Protein–C: POC) or the percentage of PON allocated to protein (Protein–N: PON) significantly and positively correlated with growth rate (R^2^=0.61 and 0.83, both *p*<0.01; Figures 6C,D).

## Discussion

In this study, we analyzed the effects of the changing carbonate chemistry on elemental contents and macromolecules of *E. huxleyi* and determined how the effects on macromolecules affect elemental contents. Although DIC, HCO_3_^−^, CO_3_^2−^, and TA concentrations and pH value were lower under the LCLpH treatment and higher under the HCHpH treatment, CO_2_, HCO_3_^−^, CO_3_^2−^, and pH were found to mainly affect the physiological and biochemical processes of *E. huxleyi* as these can be perceived by cells (Kottmeier et al., 2016; Hoppe et al., 2011). With the chosen experimental design, it is difficult to disentangle the effects of CO_2_, HCO_3_^−^, and CO_3_^2−^ on growth rate, POC, protein, and carbohydrate contents. Thus, in this study, we focused on effects of ocean carbonation and acidification on growth, elemental contents, and macromolecules. It should be noted that under the same DIC concentration, decreasing TA concentration did not significantly affect growth rate and POC content of *E. huxleyi* strain NZEH although TA ranges considered in this study are considerably narrower (Hoppe et al., 2011). Under the same TA concentration, decreasing DIC concentration or pH value significantly reduced growth rates and PIC contents of *E. huxleyi* strains NZEH, RCC1256, and RCC1266 (Hoppe et al., 2011; Zhang et al., 2021). These results suggest that the response of *E. huxleyi* to TA concentration is masked by changing DIC concentration and pH value. Therefore, DIC concentration and pH value are main factors affecting the physiological and biochemical process of *E. huxleyi*. Here, we found that elevated DIC concentration within the test range increased the total cellular carbohydrate content, which increases the contribution of carbohydrate–carbon to POC under high DIC concentration (Borchard and Engel, 2015). Ocean acidification (HCLpH) did not change the contribution of protein–carbon to POC and the contribution of protein–nitrogen to PON. Combining our results with those from other studies can help us to understand the impact of changing carbonate chemistry on physiological and biochemical metabolism of coccolithophores (Bi et al., 2018; Heidenreich et al., 2019; Feng et al., 2020).

Under high pH treatments, elevated DIC concentration in seawater facilitates a higher rate of CO_2_ diffusion into the cell, which supplies photosynthetic carbon fixation with more CO_2_ and then leads to enhanced *ETR* and growth rate of *E. huxleyi*. On the other hand, low pH value in the culture medium results in high intracellular proton concentration ([H^+^]; Dickson et al., 2007; Suffrian et al., 2011), which increases the rate of CO_2_ uptake of *E. huxleyi* (Kottmeier et al., 2016). In this case, the physiological and biochemical responses of *E. huxleyi* to low pH value are dependent on the inhibiting influence of high [H^+^] and the stimulating influence of high rate of CO_2_ uptake (Bach et al., 2015; Paul and Bach, 2020). Firstly, compared to present DIC concentration and pH value (LCHpH), low pH value in low DIC concentration (LCLpH) mainly reduces the trans-membrane H^+^ electrochemical gradient, H^+^ efflux rate, and then a high concentration of H^+^ accumulates within cells, which inhibited *ETR*, calcification and growth rate (Taylor et al., 2011; Rokitta et al., 2012). However, under ocean acidification (HCLpH), the negative effects of high H^+^ concentration on *ETR* and growth rate of *E. huxleyi* RCC1266 were offset by elevated DIC concentration. These results are also supported by other studies with different *E. huxleyi* strains (Feng et al., 2017; Jin et al., 2017; Bi et al., 2018). Secondly, under low DIC concentration, no significant difference in carbohydrate contents between low and high pH treatments indicates that high rate of CO_2_ uptake compensates for low DIC limitation on carbohydrate synthesis under low pH treatment (Nimer et al., 1994; Kottmeier et al., 2016). Furthermore, in ocean acidification condition, elevated DIC concentration downregulates the activity of microalgal carbon-concentration mechanisms (CCMs; Rost et al., 2002), and low pH value reduces calcification rates of *E. huxleyi*, which lead to less energetic cost for sustaining the operation of CCMs and calcifying. In these cases, *E. huxleyi* increases the carbohydrate or lipid contents to store more energy (Rokitta et al., 2012), and the contribution of carbohydrate–carbon to total organic carbon.

To increase growth rate, one of the most important requirements of eukaryotic phytoplankton is to increase protein synthesis rates (Sterner and Elser, 2002). Our data suggest that under high DIC concentration and high pH value (HCHpH), to maintain high growth rate, *E. huxleyi* cells produced more protein and then allocated more cellular organic carbon and nitrogen to protein. Meanwhile, under low DIC concentration, no significant differences in protein contents between low and high pH treatments could be due to the fact that to maintain intracellular pH homeostasis, *E. huxleyi* cells increase or maintain the synthesis of protein, such as the H^+^ transport protein, to compensate for low pH–induced decreases in the H^+^ efflux efficiency (Taylor et al., 2011). High protein contents and low protein activities under low pH treatment have been observed in the cyanobacterium *Trichodesmium* (Hong et al., 2017). It should be mentioned that compared to HCHpH treatment, under low DIC concentration and low pH (LCLpH) treatment, cellular protein content was significantly lower, whereas PON content was no difference, which reduced the contribution of protein–nitrogen to PON under the LCLpH treatment. These results also suggest that other nitrogen-enriched macromolecules such as ATP might contribute to PON content. To acclimate to LCLpH value, *E. huxleyi* cells increase ATP synthesis to enhance energy costs for ion transport and maintenance of intracellular pH homeostasis (Taylor et al., 2017; Lin et al., 2018).

In terms of ecological implications, the positive correlations between growth rate and *rETR*_max_ found in *E. huxleyi*, in the cyanobacterium *Microcystis aeruginosa* and *Pseudanabaena* sp. (Li et al., 2020) and in the diatom *Skeletonema costatum* (Li et al., 2021) suggest that *rETR*_max_ can be considered as a proxy for growth rate of phytoplankton and may have a potential to predict phytoplankton blooms. Furthermore, no significant differences in protein content, POC/PON ratio, and the contribution of protein–N to PON between present DIC concentration and pH value and ocean acidification treatments suggest that changing ocean carbonate chemistry in a high–CO_2_ world in future may have less influence on the nutritional quality of *E. huxleyi* (Riebesell et al., 2007).

In this study, we found that reduced pH value in the surface ocean counteracted the positive effects of elevated DIC concentration on growth rate, POC, and protein contents. Elevated DIC concentrations under both low and high pH treatments mainly increased carbohydrate contents and the contribution of carbohydrate–carbon to POC. Ocean acidification did not significantly affect the contribution of protein–carbon to POC and the contribution of protein–nitrogen to PON. Positive correlations between growth rate and the contribution of protein–carbon to POC or the contribution of protein–nitrogen to PON suggest that growth rate of *E. huxleyi* may play an important role in regulating the carbon and nitrogen allocations between biomacromolecules.

## Data Availability Statement

The original contributions presented in the study are included in the article/Supplementary Material, further inquiries can be directed to the corresponding authors.

## Author Contributions

YZ, KX, and ZL contributed to the experimental design of the study. EX, HZ, YZ, and KX performed the experiment. YZ wrote the first manuscript draft, and all authors contributed to the revisions and approved the submitted version.

## Funding

This research was funded by the National Natural Science Foundation of China (41806129 and 32001180).

## Conflict of Interest

The authors declare that the research was conducted in the absence of any commercial or financial relationships that could be constructed as a potential conflict of interest.

## Publisher’s Note

All claims expressed in this article are solely those of the authors and do not necessarily represent those of their affiliated organizations, or those of the publisher, the editors and the reviewers. Any product that may be evaluated in this article, or claim that may be made by its manufacturer, is not guaranteed or endorsed by the publisher.

## References

[ref1] Bach L. T. Mackinder L. C. M. Schulz K. G. Wheeler G. Schroeder D. C. Brownlee C.. (2013). Dissecting the impact of CO_2_ and pH on the mechanisms of photosynthesis and calcification in the coccolithophore *Emiliania huxleyi*. New Phytol. 199, 121–134. doi: 10.1111/nph.12225, PMID: 23496417

[ref2] Bach L. T. Riebesell U. Gutowska M. A. Federwisch L. Schulz K. G. (2015). A unifying concept of coccolithophore sensitivity to changing carbonate chemistry embedded in an ecological framework. Prog. Oceanogr. 135, 125–138. doi: 10.1016/j.pocean.2015.04.012

[ref3] Baker N. R. (2008). Chlorophyll fluorescence: a probe of photosynthesis *in vivo*. Annu. Rev. Plant Biol. 59, 89–113. doi: 10.1146/annurev.arplant.59.032607.092759 18444897

[ref4] Barcelos e Ramos J. Müller M. N. Riebesell U. (2010). Short-term response of the coccolithophore *Emiliania huxleyi* to an abrupt change in seawater carbon dioxide concentrations. Biogeosciences 7, 177–186. doi: 10.5194/bg-7-177-2010

[ref5] Berges J. A. Franklin D. J. Harrison P. J. (2001). Evolution of an artificial seawater medium: improvements in enriched seawater, artificial water over the past two decades. J. Phycol. 37, 1138–1145. doi: 10.1046/j.1529-8817.2001.01052.x

[ref6] Bi R. Ismar S. M. H. Sommer U. Zhao M. (2018). Simultaneous shifts in elemental stoichiometry and fatty acids of *Emiliania huxleyi* in response to environmental changes. Biogeosciences 15, 1029–1045. doi: 10.5194/bg-15-1029-2018

[ref7] Borchard C. Engel A. (2015). Size-fractionated dissolved primary production and carbohydrate composition of the coccolithophore *Emiliania huxleyi*. Biogeosciences 12, 1271–1284. doi: 10.5194/bg-12-1271-2015

[ref8] Broecker W. Clark E. (2009). Ratio of coccolith CaCO_3_ to foraminifera CaCO_3_ in late Holocene deep sea sediments. Paleoceanography 24:PA3205. doi: 10.1029/2009PA001731

[ref9] Caldeira K. Wickett M. E. (2003). Anthropogenic carbon and ocean pH–the coming centuries may see more oceanic acidification than the past 300 million years. Nature 425:365. doi: 10.1038/425365a, PMID: 14508477

[ref10] Davies B. H. (1976). “Carotenoid,” in Chemistry and Biochemistry of Plant Pigments. ed. Goodwin T. W. (New York: Academic Press), 38–65.

[ref11] Dickson A. G. Afghan J. D. Anderson G. C. (2003). Reference materials for oceanic CO_2_ analysis: a method for the certification of total alkalinity. Mar. Chem. 80, 185–197. doi: 10.1016/S0304-4203(02)00133-0

[ref12] Dickson A. G. Sabine C. L. Christian J. R. (2007). Guide to Best Practices for Ocean CO_2_ Measurements. Sidney: PICES Special Publication 3, 102–108.

[ref13] Fabry V. J. Balch W. M. (2010). “Direct measurements of calcification rates in planktonic organisms,” in Guide to Best Practices for Ocean Acidification Research and Data Reporting. eds. Riebesell U. Fabry V. J. Hansson L. Gattuso J. P. (Luxembourg: Publications Office of the European Union), 201–212.

[ref14] Feng Y. Roleda M. Y. Armstrong E. Boyd P. W. Hurd C. L. (2017). Environmental controls on the growth, photosynthetic and calcification rates of a southern hemisphere strain of the coccolithophore *Emiliania huxleyi*. Limnol. Oceanogr. 62, 519–540. doi: 10.1002/lno.10442

[ref15] Feng Y. Y. Roleda M. Y. Armstrong E. Law C. S. Boyd P. W. Hurd C. L. (2018). Environmental controls on the elemental composition of a southern hemisphere strain of the coccolithophore *Emiliania huxleyi*. Biogeosciences 15, 581–595. doi: 10.5194/bg-15-581-2018

[ref16] Feng Y. Y. Roleda M. Y. Armstrong E. Summerfield T. C. Law C. S. Hurd C. L.. (2020). Effects of multiple drivers of ocean global change on the physiology and functional gene expression of the coccolithophore *Emiliania huxleyi*. Glob. Chang. Biol. 26, 5630–5645. doi: 10.1111/GCB.15259, PMID: 32597547

[ref17] Fernie A. R. Stitt M. (2012). On the discordance of metabolomics with proteomics and transcriptomics: coping with increasing complexity in logic, chemistry, and network interactions. Plant Physiol. 158, 1139–1145. doi: 10.1104/pp.112.193235, PMID: 22253257 PMC3291261

[ref18] Geider R. J. LaRoche J. (2002). Redfield revisited: variability of C:N:P in marine microalgae and its biochemical basis. Eur. J. Phycol. 37, 1–17. doi: 10.1017/S0967026201003456

[ref19] Guillard R. R. L. Ryther J. H. (1962). Studies of marine planktonic diatoms. I. *Cyclotella nana* Hustedt and *Detonula confervacea* Cleve. Can. J. Microbiol. 8, 229–239. doi: 10.1139/m62-029, PMID: 13902807

[ref20] Heidenreich E. Wördenweber R. Kirschhöfer F. Nusser M. Friedrich F. Fahl K.. (2019). Ocean acidification has little effect on the biochemical composition of the coccolithophore *Emiliania huxleyi*. PLoS One 14:e0218564. doi: 10.1371/journal.pone.0218564, PMID: 31291290 PMC6619986

[ref21] Hong H. Shen R. Zhang F. Wen Z. Chang S. Lin W.. (2017). The complex effects of ocean acidification on the prominent N_2_-fixing cyanobacterium *Trichodesmium*. Science 356, 527–531. doi: 10.1126/science.aal2981, PMID: 28450383

[ref22] Hoppe C. J. M. Langer G. Rost B. (2011). *Emiliania huxleyi* shows identical responses to elevated pCO_2_ in TA and DIC manipulations. J. Exp. Mar. Biol. Ecol. 406, 54–62. doi: 10.1016/j.jembe.2011.06.008

[ref23] Jasby A. D. Platt T. (1976). Mathematical formulation of the relationship between photosynthesis and light for phytoplankton. Limnol. Oceanogr. 21, 540–547. doi: 10.4319/lo.1976.21.4.0540

[ref24] Jeffrey S. T. Humphrey G. F. (1975). New spectrophotometric equations for determining chlorophylls *a*, b, c1 and c2 in higher plants, algae and natural phytoplankton. Biochem. Physiol. Pflanz. 167, 191–194. doi: 10.1016/S0015-3796(17)30778-3

[ref25] Jin P. Ding J. C. Xing T. Riebesell U. Gao K. S. (2017). High levels of solar radiation offset impacts of ocean acidification on calcifying and non-calcifying strains of *Emiliania huxleyi*. Mar. Ecol. Prog. Ser. 568, 47–58. doi: 10.3354/meps12042

[ref26] Jones B. M. Iglesias-Rodriguez M. D. Skipp P. J. Edwards R. J. Greaves M. J. Young J. R.. (2013). Responses of the *Emilinia huxleyi* proteome to ocean acidification. PLoS One 8:e61868. doi: 10.1371/journal.pone.0061868, PMID: 23593500 PMC3625171

[ref27] Kondrik D. Kazakov E. Pozdnyakov D. (2019). A synthetic satellite dataset of the spatio-temporal distributions of *Emiliania huxleyi* blooms and their impacts on Arctic and sub-Arctic marine environments. Earth Syst. Sci. Data 11, 119–128. doi: 10.5194/essd-11-119-2019

[ref28] Kottmeier D. M. Rokitta S. D. Rost B. (2016). H^+^-driven increase in CO_2_ uptake and decrease in uptake explain coccolithophores’ acclimation responses to ocean acidification. Limnol. Oceanogr. 61, 2045–2057. doi: 10.1002/lno.10352

[ref29] Kubryakova E. A. Kubryakov A. A. Mikaelyan A. S. (2021). Winter coccolithophore blooms in the Black Sea: interannual variability and driving factors. J. Mar. Syst. 213:103461. doi: 10.1016/j.jmarsys.2020.103461

[ref30] Li Z. Dai G. Z. Zhang Y. Xu K. Bretherton L. Finkel Z. V.. (2020). Photosynthetic adaptation of light availability shapes the ecological success of bloom-forming cyanobacterium *Pseudanabaena* to iron limitation. J. Phycol. 56, 1457–1467. doi: 10.1111/jpy.13040, PMID: 32557638

[ref31] Li H. Xu T. Ma J. Li F. Xu J. (2021). Physiological responses of *Skeletonema costatum* to the interactions of seawater acidification and the combination of photoperiod and temperature. Biogeosciences 18, 1439–1449. doi: 10.5194/bg-18-1439-2021

[ref32] Lin Z. Wang L. Chen M. Chen J. (2018). The acute transcriptomic response of coral-algae interactions to pH fluctuation. Mar. Genomics 42, 32–40. doi: 10.1016/j.margen.2018.08.006, PMID: 30197044

[ref33] Masuko T. Minami A. Iwasaki N. Majima T. Nishimura S. I. Lee Y. C. (2005). Carbohydrate analysis by a phenol-sulfuric acid method in microplate format. Anal. Biochem. 339, 69–72. doi: 10.1016/j.ab.2004.12.001, PMID: 15766712

[ref34] Meyer J. Riebesell U. (2015). Reviews and syntheses: response of coccolithophores to ocean acidification: a meta-analysis. Biogeosciences 12, 1671–1682. doi: 10.5194/bg-12-1671-2015

[ref35] Monteiro F. M. Bach L. T. Brownlee C. Bown P. Rickaby R. E. M. Poulton A. J.. (2016). Why marine phytoplankton calcify. Sci. Adv. 2:e1501822. doi: 10.1126/sciadv.1501822, PMID: 27453937 PMC4956192

[ref36] Monteiro C. M. Castro M. L. Malcata F. X. (2009). Use of the microalga *Scenedesmus obliquus* to remove cadmium cations from aqueous solutions. World J. Microbiol. Biotechnol. 25, 1573–1578. doi: 10.1007/s11274-009-0046-y

[ref37] Müller M. N. Beaufort L. Bernard O. Pedrotti M. L. Talec A. Sciandra A. (2012). Influence of CO_2_ and nitrogen limitation on the coccolith volume of *Emiliania huxleyi* (Haptophyta). Biogeosciences 9, 4155–4167. doi: 10.5194/bg-9-4155-2012

[ref38] Ni G. Zimbalatti G. Murphy C. D. Barnett A. B. Arsenault C. M. Li G.. (2016). Arctic *micromonas* uses protein pools and non-photochemical quenching to cope with temperature restrictions on photosystem II protein turnover. Photosynth. Res. 131, 203–220. doi: 10.1007/s11120-016-0310-6, PMID: 27639727 PMC5247552

[ref39] Nimer N. A. Brownlee C. Merrett M. J. (1994). Carbon dioxide availability, intracellular pH and growth rate of the coccolithophore *Emiliania huxleyi*. Mar. Ecol. Prog. Ser. 109, 257–262. doi: 10.3354/meps109257

[ref40] Pakulski J. D. Benner R. (1992). An improved method for the hydrolysis and MBTH analysis of dissolved and particulate carbohydrates in seawater. Mar. Chem. 40, 143–160. doi: 10.1016/0304-4203(92)90020-B

[ref41] Paul A. J. Bach L. T. (2020). Universal response pattern of phytoplankton growth rates to increasing CO_2_. New Phytol. 228, 1710–1716. doi: 10.1111/nph.16806, PMID: 32654139

[ref42] Pierrot D. Lewis E. Wallace D. W. R. (2006). MS Excel Program Developed for CO_2_ System Calculations, ORNL/CDIAC-105. Oak Ridge, Tenn: Carbon Dioxide Information Analysis Centre, Oak Ridge National Laboratory, U.S., Department of Energy.

[ref43] Poulton A. J. Adey T. R. Balch W. M. Holligan P. M. (2007). Relating coccolithophore calcifcation rates to phytoplankton community dynamics: regional diferences and implications for carbon export. Deep-Sea Res. II Top. Stud. Oceanogr. 54, 538–557. doi: 10.1016/j.dsr2.2006.12.003

[ref44] R Core Team (2018). The R foundation for statistical computing platform: x86_64-w64-mingw32/x64. Available at: https://cran.r-project.org/bin/windows/base/old/3.5.0 (Accessed: May 20, 2021).

[ref45] Ralph P. J. Gademann R. (2005). Rapid light curves: A powerful tool to assess photosynthetic activity. Aquat. Bot. 82, 222–237. doi: 10.1016/j.aquabot.2005.02.006

[ref46] Riebesell U. Schulz K. G. Bellerby R. G. J. Botros M. Fritsche P. Meyerhöfer M.. (2007). Enhanced biological carbon consumption in a high CO_2_ ocean. Nature 450, 545–549. doi: 10.1038/nature06267, PMID: 17994008

[ref47] Riebesell U. Tortell P. D. (2011). “Effects of ocean acidification on pelagic organisms and ecosystems,” in Ocean Acidification. eds. Gattuso J. P. Hansson L. (Oxford: Oxford University Press), 99–121.

[ref48] Rokitta S. D. John U. Rost B. (2012). Ocean acidification affects redox-balance and ion-homeostasis in the life-cycle stages of *Emiliania huxleyi*. PLoS One 7:e52212. doi: 10.1371/journal.pone.0052212, PMID: 23300616 PMC3530605

[ref49] Rost B. Zondervan I. Riebesell U. (2002). Light-dependent carbon isotope fractionation in the coccolithophorid *Emiliania huxleyi*. Limnol. Oceanogr. 47, 120–128. doi: 10.4319/lo.2002.47.1.0120

[ref50] Roy R. N. Roy L. N. Vogel K. M. Porter-Moore C. Pearson T. Good C. E.. (1993). Thermodynamics of the dissociation of boric acid in seawater at S 5 35 from 0 degrees C to 55 degrees C. Mar. Chem. 44, 243–248. doi: 10.1016/0304-4203(93)90206-4

[ref51] Sterner R. W. Elser J. J. (2002). Ecological Stoichiometry: The Biology of Elements From Molecules to the Biosphere. Princeton: Princeton University Press, 80–133.

[ref52] Suffrian K. Schulz K. G. Gutowska M. A. Riebesell U. Bleich M. (2011). Cellular pH measurements in *Emiliania huxleyi* reveal pronounced membrane proton permeability. New Phytol. 190, 595–608. doi: 10.1111/j.1469-8137.2010.03633.x, PMID: 21294736

[ref53] Taylor A. R. Brownlee C. Wheeler G. (2017). Coccolithophore cell biology: chalking up progress. Annu. Rev. Mar. Sci. 9, 283–310. doi: 10.1146/annurev-marine-122414-034032 27814031

[ref54] Taylor A. R. Chrachri A. Wheeler G. Goddard H. Brownlee C. (2011). A voltage-gated H^+^ channel underlying pH homeostasis in calcifying coccolithophores. PLoS Biol. 9:e1001085. doi: 10.1371/journal.pbio.1001085, PMID: 21713028 PMC3119654

[ref55] Tyrell T. Merico A. (2004). “*Emiliania huxleyi*: bloom observations and the conditions that induce them,” in Coccolithophores: From Molecular Biology to Global Impact. eds. Thierstein H. R. Young J. (Berlin: Springer), 75–98.

[ref56] Zhang Y. Bach L. T. Schulz K. G. Riebesell U. (2015). The modulating effect of light intensity on the response of the coccolithophore *Gephyrocapsa oceanica* to ocean acidification. Limnol. Oceanogr. 60, 2145–2157. doi: 10.1002/lno.10161

[ref57] Zhang Y. Li Z. K. Schulz K. G. Hu Y. Irwin A. J. Finkel Z. V. (2021). Growth-dependent changes in elemental stoichiometry and macromolecular allocation in the coccolithophore *Emiliania huxleyi* under different environmental conditions. Limnol. Oceanogr. 66, 2999–3009. doi: 10.1002/lno.11854

