# Supplementary material for: Disentangling the Effects of Ocean Carbonation and Acidification on Elemental Contents and Macromolecules of the Coccolithophore Emiliania huxleyi
Source: Front Microbiol. 2021 Oct 20;12:737454. doi: 10.3389/fmicb.2021.737454 (PMC8564145; doi:10.3389/fmicb.2021.737454)
Supplement: Supplementary file 1 [file Data_Sheet_1.PDF]

TABLE S1 | Summary of initial and final cell abundances of each pre–culture, and calculation of acclimating generations in each treatment during the pre–cultures.

	LCHpH (Present DIC and pH)	LCLpH (Reduced pH)	HCHpH (Ocean carbonation)	HCLpH (Ocean acidification)
Initial cell abundance (cell ml^{-1})	3040±56	3080±80	3010±105	3150±67
Final cell abundance (cell ml^{-1})	43300±1590	32420±1240	67350±1830	41490±960
Increasing fold	14.2±0.7	10.5±0.3	22.3±0.9	13.2±0.3
Acclimating generation during 2 days incubation	3.8±0.1	3.4±0.1	4.4±0.1	3.7±0.1
Acclimating generation during 8 days incubation	15.3	13.6	17.9	14.9

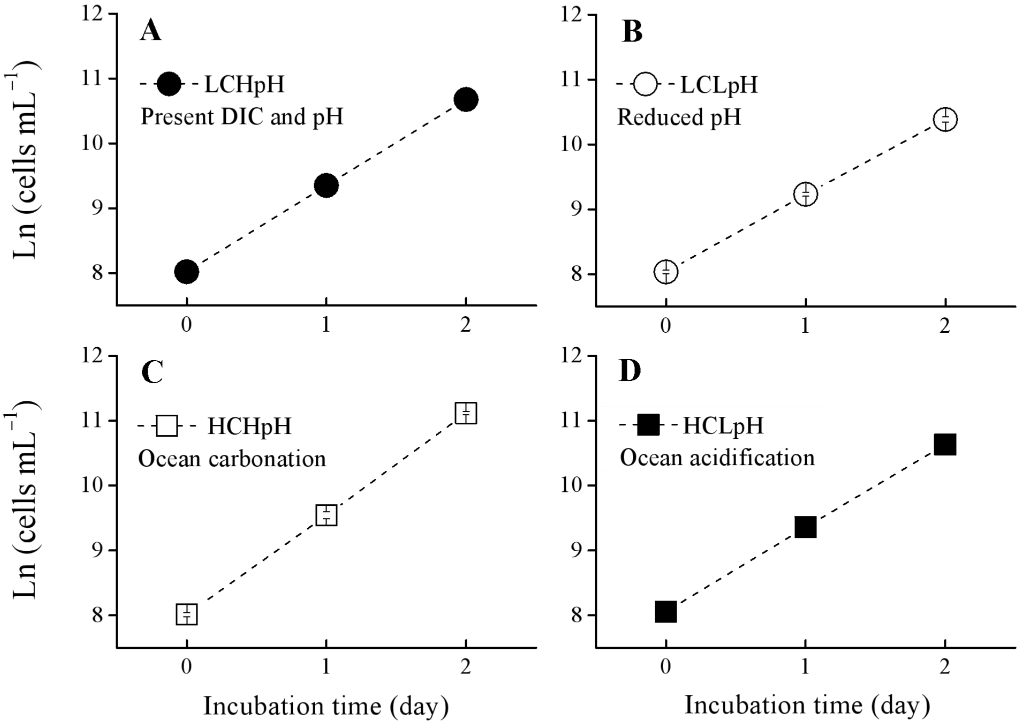

FIGURE S1 | Cell density of *E. huxleyi* RCC1266 under **(A)** the present DIC and pH (LCHpH), **(B)** the low DIC and low pH (LCLpH, reduced pH), **(C)** the high DIC and high pH (HCHpH, ocean carbonation), and **(D)** the high DIC and low pH (HCLpH, ocean acidification) treatments. The data represents the means and standard deviation (sd) of four independent cultures.

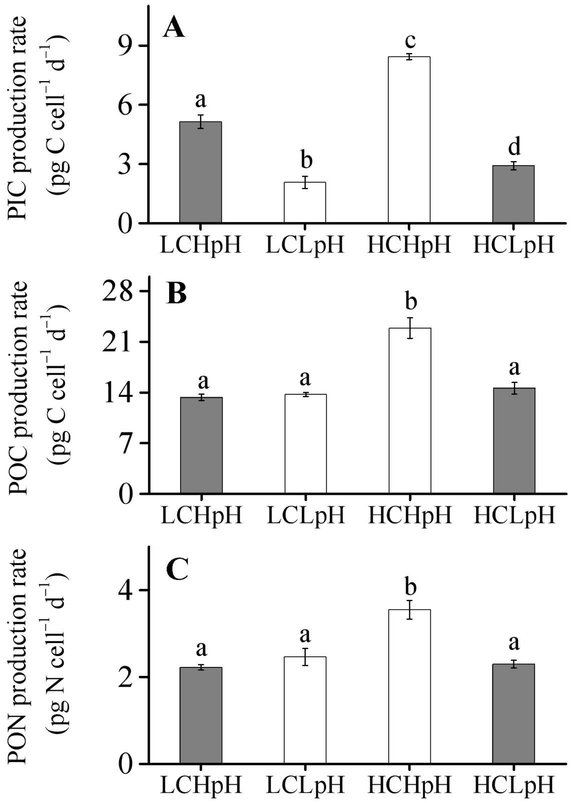

FIGURE S2 | (A) Particulate inorganic carbon (PIC), **(B)** particulate organic carbon (POC), and **(C)** particulate organic nitrogen (PON) production rates of *E. huxleyi* RCC1266 under the LCHpH (gray), LCLpH, HCHpH, and HCLpH (gray) treatments. Different letters (a, b, c, d) in each panel represent significant differences between different treatments (Tukey Post hoc, $p < 0.05$). For more information, please see figure 1.
